# Supplementary material for: Assistive technology use in domestic activities by people who are blind
Source: Sci Rep. 2025 Mar 3;15:7486. doi: 10.1038/s41598-025-91755-w (PMC11876317; doi:10.1038/s41598-025-91755-w)
Supplement: Supplementary file 1 — Supplementary Information. [file 41598_2025_91755_MOESM1_ESM.pdf]

Interview Questions

Demographics

- Name
- Age
- How long have you been a student at the SB Braille Institute and/or with Blind Fitness?
    - 0-10 years, 10-20 years, 20 + years

Blindness level

- When did you become blind?
- How long have you been blind?
- How did you become blind?
    - What was the timeline?
- What is your level of blindness?
- Do you have the same level of vision in each eye?

Daily life

- Walk me through a normal day for you. What does your schedule look like, and what do you do on a daily basis?
- Which of the following tools/resources do you use to support your blindness?
    - Canes, guide dog, personal guide, family, and/or friends?
- Do you live with anyone?
    - How often are they around?
    - How long have you lived with them?
    - Do they help you in day-to-day tasks?

Assistive tool and technology use

- Which of the following smart technologies do you use in your day-to-day life to assist you with different tasks?
    - *General:* Seeing AI, Be My Eyes, Google Lookout, TapTapSee, iDentifi, ThirdEye, BeSpecular, ViaOpta, Navilens, Travelear, Envision
    - *Magnifiers:* Smart Magnifier
    - *Navigation:* Soundscape, Intersection Explorer, BlindSquare, Lazarillo, Arianna, Lazzus
    - *Guided GPS systems:* Microsoft 3D sound maps, Guide Dog GPS
    - *Reading:* Google Text-to-Speech
    - *Cinema:* Greta
    - *Devices:* Feelware, Sunu, Fingerreader, Braille ebook reader, OrCam, eSight, nuEyes, Aira, iBionics, visual prosthetic devices
    - *Tactile labeling:* wikki stix, bump dots, braille labeller
    - *Other*
- What is your comfort level/proficiency in each of these different technologies?
    - 1. Easy
    - 2. Moderate
    - 3. Difficult
    - 4. Impossible
- What are your general thoughts about existing assistive technologies?
    - Tech they know about but don’t use - why not?
- What complaints do you have about existing assistive technologies, if any?

- How did they find out about these technologies that you use?
    - Braille institute, family/friends/word of mouth, internet, and/or other?

Activities of daily living

- How would you say your general life habits have changed, if at all, since becoming blind?
- What is the most frustrating thing about being blind, if anything?
- Which tasks do you find to be the most difficult?
- On a scale of 1-5, how difficult is it for you to do iADLs by yourself (while using the technology/tools you currently use for these tasks)?
    - If someone else does the task for them, how difficult would it be for you to do the task if they had to learn how to do it alone?
    - What is your strategy for the various iADLs?
    - Which technologies do you use for each of these tasks (on your smartphone or other devices)?
    - Do you use other tools for any of these tasks, such as tactile labeling, requesting human assistance, or using other sensory information?
    - What are some challenges you face when doing these tasks?
